# ALK5 deficiency inhibits macrophage inflammation and lipid loading by targeting KLF4

**DOI:** 10.1042/BSR20194188

**Published:** 2020-03-04

**Authors:** Wenyan Li, Junhua Wang, Zhaofeng Li

**Affiliations:** The First Hospital of Nanchang, Nanchang, 330008, China

**Keywords:** ALK5, foam cell, inflammation, KLF4, macrophage

## Abstract

The transforming growth factor type-β (TGF-β) has been demonstrated to play an important role in the development of atherosclerosis through binding to the serine/threonine kinase transmembrane type I and type II receptors. However, as a key type I receptor for TGF-β, the exact role and the underlying mechanism of Activin receptor-like kinase 5 (ALK5) on macrophage activation involved in atherogenesis remain unclear. In the present study, enhanced ALK5 expression was found in bone marrow derived macrophages (BMDMs) upon OX-LDL stimulation tested by RT-PCR and Western blot, which was further verified by co-immunofluorescence staining. Next, the loss-of-function of ALK5 used AdshALK5 transfection was performed to test the effect of ALK5 on macrophage activation. We observed that ALK5 silencing inhibited pro-inflammatory but promoted anti-inflammatory macrophage markers expression. Moreover, decreased foam cell formation was found in ALK5 knockdown macrophages accompanied by increased cholesterol efflux. Mechanistically, ALK5 knockdown significantly increased KLF4 expression that was responsible for the attenuated macrophage activation induced by ALK5 knockdown. Collectively, these findings suggested that neutralization of ALK5 may act as a promising strategy for the management of atherosclerosis.

## Introduction

Atherosclerosis is well recognized as a chronic inflammatory disease [1] and accounts for the development of cardiovascular diseases (CVDs) that is the leading cause of mortality and morbidity worldwide, including myocardial infarction, sudden cardiac death and stroke [2]. During the development of atherosclerosis, the recruited and infiltrated monocyte-macrophage subendothelium initiated by endothelial dysfunction play an important role in the whole phrase of atherogenesis [3]. Notably, macrophage has emerged to show plastic properties and can regulate inflammatory response by shifting the activation of classically pro-inflammatory M1 macrophages and resolving M2 macrophages [4]. More importantly, the excess engulfment of oxidized low-density lipoprotein gives rise to foam cell formation and accelerates atherogenesis associated with the vulnerable plaques [5]. Therefore, exploring the key regulator of macrophage activation implicated in atherogenesis may provide an effective strategy for preventing atherosclerosis.

The present study exhibited that ALK5 expression was significantly up-regulated in BMDMs with OX-LDL administration and primary located in cytoplasm of macrophages. Loss-of-function study demonstrated that ALK5 silencing dramatically inhibited inflammatory response and foam cell formation. Mechanistically, we found that ALK5 knockdown significantly promoted KLF4 expression and the effect of ALK5 silencing on macrophage activation was largely reversed by KLF4 inactivation.

## Methods and materials

TGF-β family members are implicated in the regulation of growth control, positional information and cardiac organogenesis, which has emerged to play an important role in cardiovascular diseases [6]. In which, members of TGF-β family of cytokines signal exert its functional role via serine/threonine kinase transmembrane complex receptor contained type I and type II receptors [6]. ALK members act as the key type I receptors that can be activated by binding TGF-β to the receptor complex and initiate the key downstream signaling involving Smad transcription factors, mitogen-activated protein kinases (MAPK) and PI3K-Akt signaling [7]. Among them, ALK5 is demonstrated to be widely expressed in most tissues and cell types, and participates in various diseases. ALK5 reveals a distinct role involved in regulation of heart development [8] and vascular development [9]. The ALK5 mediated TGF-β-induced ET-1 [10] and activation phosphorylation of Smad2/3 [11] play an important role in migration and proliferation of endothelial cells, meanwhile ALK5 also phosphorylates the endogen cytoplasmic domain to activate Smad1/5/8 signaling leading to increased migration of endothelial cell [12]. ALK5 is responsible for TGF-β-inducted smooth muscle cell differentiation and proliferation within familial pulmonary arterial hypertension [13]. Additionally, ALK5 has an effect on fibrotic disease [14] and implicated in multiple tumor development [15]. However, the specific effect of ALK5 on macrophage activation implicated in atherogenesis is unexplored.

### Cell culture and infection with recombinant adenoviral vectors

The 8- to10-week-old ApoE deficiency mice received intraperitoneal anesthesia with pentobarbital sodium (50 mg/kg), then were killed in random order. Approximately 5 × 10^7^ nucleated bone marrow cells from the femurs and tibias of each mouse were collected and then cultured in 10 ml of RPMI with 10% fetal bovine serum and MCSF (50 ng/ml). To knockdown ALK5 expression, ALK5 specific short hairpin RNA (shRNA)-expressing (shALK5) construction was mediated by pENTR/U6-shRNA vector and homologous recombination of adenovirus skeleton plasmid, while adenoviral short hairpin RNA (AdshSCR) served as controls. The BMDMs were transfected with the above adenovirus according to the manufacturer’s protocol and treated with OX-LDL at the individual concentration (25 or 50 ng/ml). The animal study protocols were performed according to Guide for the Care and Use of Laboratory Animals published by the US National Institutes of Health. The Animal Care and Use Committee of The First Hospital of Nanchang approved all study protocols.

### Quantitative real-time PCR

Total mRNA was isolated using TRIzol reagent and extracted from cultured BMDMs. Subsequently, the mRNA was performed for reversely transcription into cDNA with a Transcriptor First Strand cDNA Synthesis Kit (Roche, Basel) according to the manufacturer’s protocol. First-strand cDNA was subjected to real-time PCR with SYBR green on a LightCycler 480 QPCR System (Roche Diagnostics). Gene expression was normalized to GAPDH.

### Western blot

Western blots were performed following standard protocols as previous described [16]. Briefly, the proteins were separated by SDS-PAGE and then transferred to polyvinylidene fluoride (PVDF) membranes, which were probed with the appropriate primary antibodies overnight at 4°C. Then, the membranes were incubated with the appropriate secondary antibodies before visualization by chemiluminescence with Molecular Imager ChemiDoc TM XRS+ using Image Lab TM Software 5.1 (Bio-Rad, Bio-Legend Scientific Co. Ltd). Protein expression was normalized to GAPDH.

### Immunofluorescence staining

The BMDMs were fixed with 3.7% formaldehyde in PBS for 15 min at room temperature and permeabilized with 0.1% Triton X-100 in PBS for 40 min. After incubating with primary antibodies at 4°C overnight, the slices were rewarmed at 37°C for half an hour and washed with PBS before incubation with appropriate fluorescence-labeled secondary antibody for 1 h. DAPI was performed for cell nuclei detection.

### Statistical analysis

Data were represented as the means ± SD. Data were analyzed with a two-tailed Student’s *t* test or one-way ANOVA followed by a Bonferroni post hoc test or Tamhane’s T2 post hoc test. All statistical analyses were performed using SPSS, version 22.0. Differences were considered significant at a value of *P* < 0.05.

## Results

### ALK5 expression is enhanced in activated macrophage

**Figure 1:**
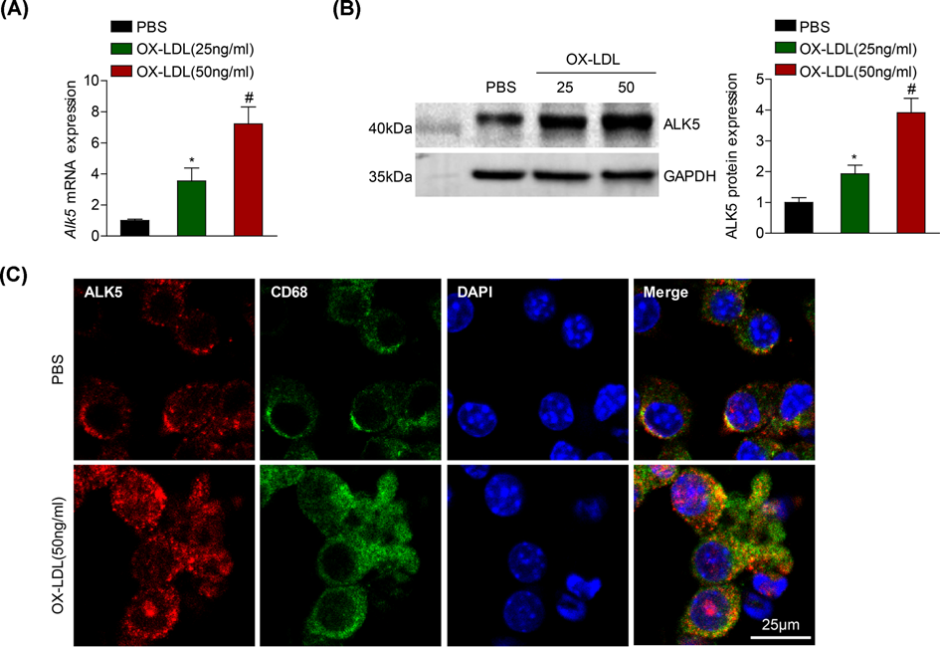
Increased ALK5 expression in macrophage induced by OX-LDL (**A** and **B**) RT-PCR analysis or Western blot analysis of ALK5 mRNA (A) or protein (B) expression in BMDMs after 24 h of stimulation with OX-LDL at the indicated concentration (25 and 50 ng/ml); *n* = 3. (**C**) Co-staining of ALK5 expression in macrophage with ALK5 (red) and CD68 (green) induced by OX-LDL (50 ng/ml); *n* = 3; scale bar = 25 μm. *P < 0.05 compared with PBS treated group. #*P* < 0.05 compared with OX-LDL (25 ng/ml) treated group.

To explore the effect of ALK5 on macrophage activation involved in the development of atherosclerosis, we first examined whether ALK5 expression was changed in isolated BMDMs upon OX-LDL administration. RT-PCR analysis revealed that *Alk5* mRNA expression was dramatically up-regulated in BMDMs treated with OX-LDL in a manner following increased OX-LDL concentration compared with the baseline level obtained with PBS treatment (Figure 1A). As expected, the ALK5 expression in mRNA level was further verified in the protein level tested by Western blot analysis (Figure 1B). Continued, we observed the increased ALK5 expression was primarily localized in the cytoplasm of macrophage followed by OX-LDL stimulation by co-immunofluorescence staining with ALK5 and macrophage-specific marker (Figure 1C). The above results suggested that ALK5 was up-regulated and most located in activated macrophage.

### AKL5 knockdown suppresses classical polarized macrophages and foam cell formation

**Figure 2:**
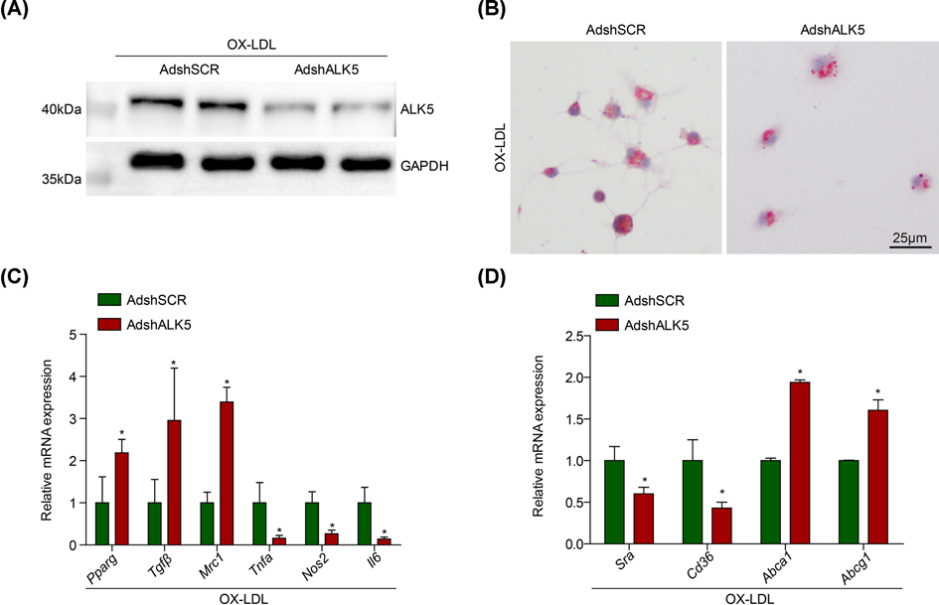
ALK5 knockdown inhibits macrophage activation (**A**) Western blot analysis of ALK5 level in BMDMs transfected with AdshALK5 or AdshSCR upon OX-LDL (50 ng/ml) treatment; *n* = 3. (**B**) Attenuated M1 markers but enhanced M2 markers gene expression in BMDMs infected with AdshALK5 or AdshSCR induced by OX-LDL; *n* = 3. (**C**) Foam cell formation of macrophages transfected with AdshALK5 or AdshSCR induced by OX-LDL; scale bar = 25 μm; *n* = 9. (**D**) RT-PCR analysis of *Cd36, Sra, Abca1* and *Abcg1* expression in BMDMs transfected with AdshALK5 or AdshSCR upon OX-LDL administration; *n* = 3. *P < 0.05 compared with control group.

Considering that ALK5 expression was induced in activated macrophages with OX-LDL treatment, the BMDMs infected with adenovirus harboring ALK5 short hairpin RNA (AdshALK5) were utilized to investigated the possible regulation of ALK5 on macrophage inflammation and foam cell formation implicated in atherogenesis. The efficiency of ALK5 knockdown in BMDMs was verified with Western blot analysis (Figure 2A). The balance of pro- or anti- inflammation mediated by macrophages plays an important role in atherogenesis, we noticed that the mRNA levels of pro-inflammatory M1 macrophage markers were significantly decreased by ALK5 knockdown compared with AdshSCR group, including *Tnfα, Nos2* and *Il6* (Figure 2B). By contrast, the mRNA levels of alternative M2 macrophage markers were increased by ALK5 knockdown, including *Pparg, Tgfβ* and *Mrc1* (Figure 2B). The uptake of oxidized low-density lipoprotein leads to foam cell formation acts as the critical process in atherogenesis. The foam cell formation in BMDMs infected with AdshALK5 was decreased exanimated with oil red O for neutral lipid staining compared with AdshSCR group (Figure 2C). Additionally, we observed that the mRNA levels of *Cd36* and *Sra* were decreased, whereas the *Abca1* and *Abcg1* were increased in BMDMs infected with AdshALK5 (Figure 2D).

### ALK5 knockdown promotes KLF4 expression

**Figure 3:**
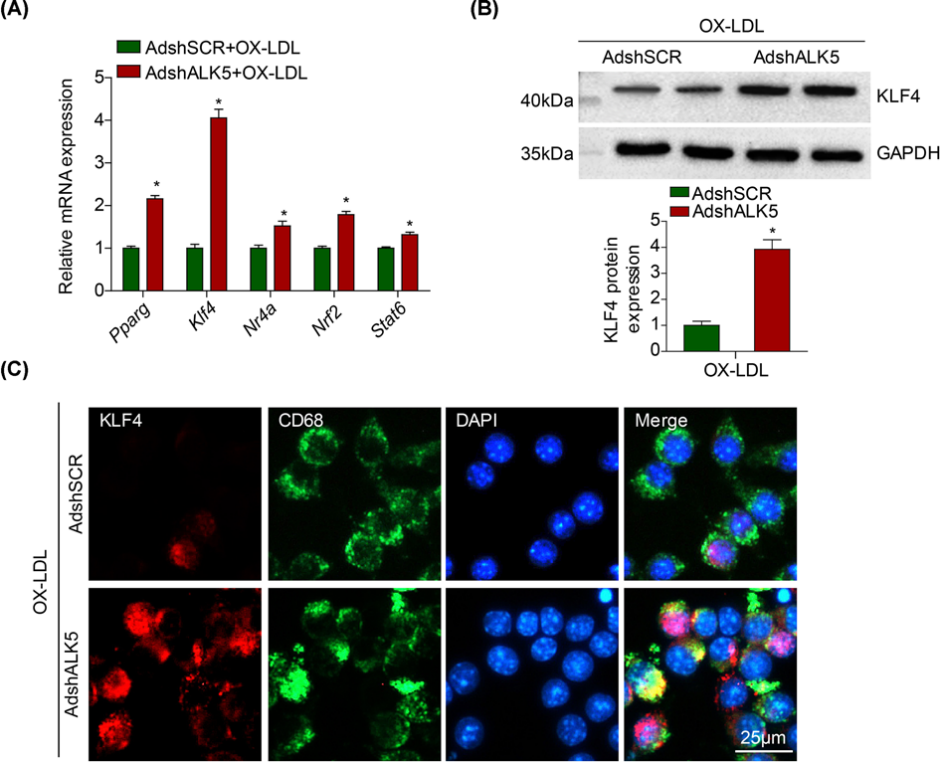
Enhanced KLF4 expression in macrophages of ALK5 knockdown (**A**) RT-PCR analysis of regulator genes in macrophages of ALK5 knockdown or AdshSCR induced by OX-LDL; *n* = 3. (**B**) Western blot analysis of KLF4 in macrophages of AdshALK5 or AdshSCR induced by OX-LDL; *n* = 3. (**C**) Double-immunofluorescence staining of KLF4 (red) in macrophages (CD68, green) transfected with AdshALK5 or AdshSCR upon OX-LDL stimulation; scale bar = 25 μm; *n* = 5; *P < 0.05 compared with control group.

Dozens of evidences have demonstrated that the regulation of macrophage polarization and foam cell are controlled by various genes, the represented markers including *Pparg, Klf4, Nr4a, Nrf2* and *Stat6* [17]. The results in our study exhibited that KLF4 expression is the most significantly changed targets in BMDMs transfected with AdshALK5 with OX-LDL treatment compared with AdshSCR group, which has reported to play an important role in atherogenesis (Figure 3A). Consistent with the observation in mRNA level, we also noticed that KLF4 protein expression was enhanced in BMDMs of ALK5 knockdown compared with AdshSCR group (Figure 3B). The co-immunofluorescence staining further demonstrated that increased KLF4 expression was primarily localized in the nuclei of macrophages upon OX-LDL administration in AdshALK5 group compared with AdshSCR group (Figure 3C).

### KLF4 is required for the effect of ALK5 on macrophage activation

**Figure 4:**
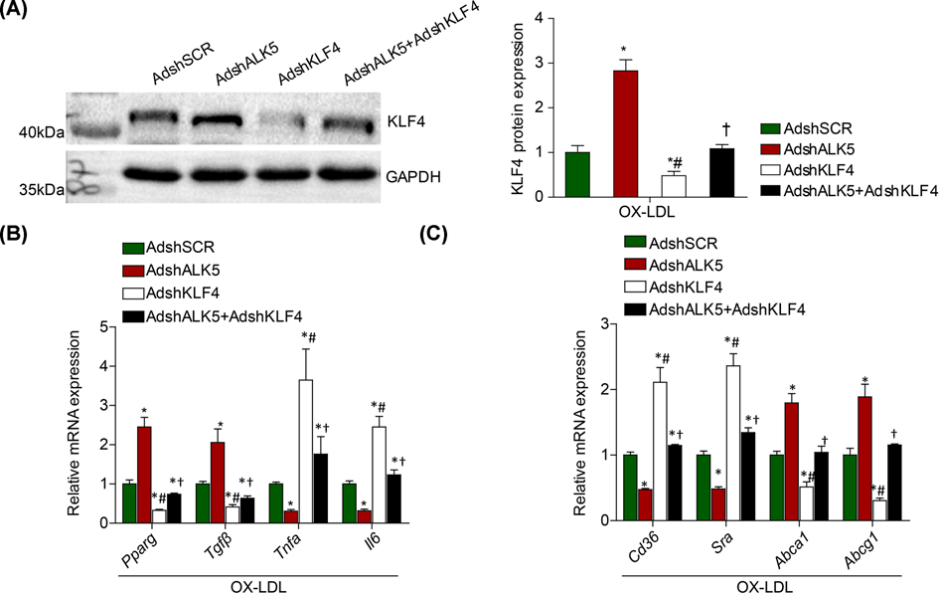
The effect of ALK5 silencing on macrophage activation is mediated by KLF4 (**A**) Western blot analysis of KLF4 in BMDMs infected with AdshSCR, AdshALK5, AdshKLF4 and AdshALK5+AdshKLF4; *n* = 3. (**B**) RT-PCR analysis of M1 and M2 macrophage markers gene expression in BMDMs co-infected with AdshSCR, AdshALK5, AdshKLF4 and AdshALK5+AdshKLF4; *n* = 3. (**C**) RT-PCR analysis of CD36 and SR-A, as well as ABCA-1 and ABCG-1 in BMDMs co-infected with AdshSCR, AdshALK5, AdshKLF4 and AdshALK5+AdshKLF4; *n* = 3. *P < 0.05 compared with AdshSCR group. #*P* < 0.05 compared with AdshALK5 group. †*P* < 0.05 compared with AdshKLF4 group.

Next, we transfect BMDMs with AdshKLF4 to test the functional regulation of KLF4 knockdown on the effect of macrophage polarization and foam cell formation mediated by ALK5 knockdown. We observed a significant down-regulation of KLF4 expression in BMDMs infected with KLF4 knockdown followed with OX-LDL treatment (Figure 4A). Importantly, we found the increased ratio of anti-inflammatory M2 markers to pro-inflammatory M1 markers (Figure 4B), as well as the accompanied high ratio of cholesterol efflux to uptake (Figure 4C), mediated by ALK5 knockdown were significantly reversed by KLF4 inactivation. On the basis of the observation, we concluded that KLF4 was the important downstream target for the effect of macrophage activation regulated by ALK5 silencing.

## Discussion

In the present study, we first demonstrated that an up-regulation of ALK5 expression was found in activated macrophage and co-located in cytoplasm with OX-LDL administration. The loss-of-function of ALK5 *in vitro* attenuated pro-inflammatory but promoted anti-inflammatory macrophage markers expression. Moreover, ALK5 silencing inhibited foam cell formation accompanied by increased cholesterol efflux but decreased cholesterol uptake. Furthermore, KLF4 expression was accelerated by ALK5 knockdown and the down-regulated KLF4 largely reversed the effect of ALK5 silencing on macrophage activation. On the basis of the results, we exhibited a novel role of ALK5 on macrophage activation involved in atherogenesis that was partially through attenuated KLF4 activation.

Atherosclerosis is recognized as a complex multifactorial process and a chronic inflammatory disease contributed to severe cardiovascular event, which can be triggered by various stimuli [18]. Among them, TGF-β as a represented immunomodulatory cytokine is of particular interest to cardiovascular biologists through regulation of multiple cell types involved in blood vessel wall [6]. Notably, accumulative evidences have demonstrated that TGF-β exerts an important role in atherogenesis [19,20], whereas the studies of TGF-β receptor modulation account for the effect remains unclear. Members of the TGF-β family of cytokines signal exert the functional regulation of the pathological process via serine/threonine kinase transmembrane type I and type II receptors at the cell membrane [6]. Binding TGF-β to the receptor complex triggers activation of type I receptor terms as ALK, which can initiate downstream signaling including Smad phosphorylation, MAPK and PI3K-Akt signaling, while all three cascades have been implicated in the development of atherosclerosis [19]. Previous studies have demonstrated that ALK5 is widely expressed multiple cell types involved in blood vessels system, and exerts an important role in regulation of vascular cells behavior and function implicated in various diseases. Enhanced ALK5 expression was found and localized in arterial endothelium, vascular smooth muscle cell in the medial and adventitial layers of blood vessels [9]. Functionally, ALK5-null embryos exhibit a defect in the formation of vascular smooth muscle layers, whereas the properly generation of lumens of blood vessels during embryonic development [9]. TGF-β-mediated ALK5 activation induces phosphorylation of Smad2 and 3 resulting in inhibition of endothelial cells proliferation *in vitro* [11], while the induction of ET-1 by the signaling also play an important role in regulation of migration and proliferation of endothelial cells [10]. ALK5 is required for shear stress-mediated KLF2 expression [21] and TGF-β-induced pulmonary endothelial permeability [22]. Moreover, ALK5 mediates abnormal proliferation of vascular smooth muscle cells from patients with familial pulmonary arterial hypertension [13] and is required for TGF-β-induction of smooth muscle cell differentiation markers through Smad3 and MAPK pathway [23]. Although the important role of ALK5 in endothelial cell and smooth muscle cell has been explored, the expression and function of ALK5 on macrophage has not been deeply investigated. In our study, ALK5 expression was found to show significant up-regulation in BMDMs with OX-LDL administration and primary located in cytoplasm of macrophages. Accumulative evidences have demonstrated that TGF-β1/ALK5 signaling induces monocyte migration through PI3K and P38 pathways [24], while inhibition of ALK5 promotes M2 marker expression but decreases the acquisition of M1 polarization markers [25]. Consistent with the evidences, our current study demonstrated that ALK5 knockdown increased M2 but attenuated M1 polarized macrophages.

In seeking the effectively underlying mechanism mediated of the effect of macrophage polarization switching and foam cell formation in BMDMs transfected with AdshSCR, we performed RT-PCR analysis of the represented regulator involved in macrophage activation in atherogenesis and the result suggested that KLF4 was the markedly altered target. KLF4 acts as a novel important regulator for the macrophage behaviors implicated in the chronic inflammatory diseases, especially atherosclerosis [26,27]. It has been demonstrated that KLF4 was markedly induced in response to TNF-α or LPS, and decreased by TGF-β1 in macrophages [28]. Additionally, Jain et al. reported that KLF4 expression in macrophage was robustly induced in M2 macrophages and strongly reduced in M1 macrophages [29]. In the present study, downregulated ALK5 dramatically increased KLF4 expression in mRNA and protein level, and specific in the nucleus of macrophages. Besides, KLF4 was found to cooperate with Stat6 to induce an M2 genetic program and inhibit M1 targets via sequestration of coactivators required for NF-κB activation, which suggested KLF4 to be a novel regulator of macrophage polarization [29] and the treatment of atherosclerosis [26,27]. The present study demonstrated that ALK5 knockdown promoted alternative M2 macrophage but inhibited classic M1 macrophage, while the KLF4 silencing significantly reversed the process which consistent with the previous studies. Thus, we speculated the ALK5 knockdown attenuated macrophage activation, especially macrophage polarization, in part by up-regulating KLF4 expression that may play an important role in against atherogenesis.

In conclusion, we demonstrated that the down-regulated ALK5 expression in macrophage significantly inhibited M1 but promoted M2 polarized macrophages. Additionally, ALK5 knockdown attenuated foam cell formation characterized as enhanced cholesterol efflux than uptake. The up-regulation of KLF4 expression was partially responsible for the protective role of ALK5 silencing on macrophage activation. Our current study provided that neutralization of ALK5 may act as a promising therapeutic approach for the treatment of atherosclerosis.

## Abbreviations

ALK5: activin receptor-like kinase 5
BMDM: bone marrow derived macrophage
CVD: cardiovascular disease
TGF-β: transforming growth factor type-β
